# Brucellosis presenting as piriformis myositis: a case report

**DOI:** 10.1186/1752-1947-5-125

**Published:** 2011-03-30

**Authors:** Pantelis Kraniotis, Markos Marangos, Alexandra Lekkou, Odysseas Romanos, Ekaterini Solomou

**Affiliations:** 1 Department of Radiology, University Hospital of Patras, Rion, Greece; 2 Department of Internal Medicine, Division of Infectious Diseases, University Hospital of Patras, Rion, Greece; 3 Department of Internal Medicine, University Hospital of Patras, Rion, Greece

## Abstract

**Introduction:**

Myositis is a rare bacterial muscle infection. Involvement of the piriformis muscle has been rarely reported in the literature. In this report we describe a case of piriformis myositis due to *Brucella melitensis*, which to the best of our knowledge is the first such case presented in the literature.

**Case presentation:**

We report the case of a 19-year-old Caucasian man who presented to our institution with fever and right hip pain. Brucellosis was suspected, but the clinical suspicion was for spondylodiscitis. A pelvic magnetic resonance imaging scan allowed prompt diagnosis of inflammatory involvement of the right piriformis muscle. Blood culture results were positive for *B. melitensis*. Our patient was treated with antibiotics, and follow-up magnetic resonance imaging scans showed resolution of the inflammation.

**Conclusion:**

Brucellosis can present as piriformis myositis. The clinical diagnosis of piriformis myositis is difficult, as it can mimic other common entities such as referred back pain from spondylodiscitis. Magnetic resonance imaging is the method of choice for establishing the diagnosis in the early stages of the disease, as late diagnosis can lead to abscess formation and the need for drainage.

## Introduction

Myositis is a rare muscle infection, with the most commonly implicated bacteria being *Staphylococcus *and *Streptococcus*. Piriformis myositis has been rarely reported in the literature [1-5]. Recognized predisposing factors for the condition are mainly previous viral or parasitic infections, rheumatic disease and HIV infection [6]. The clinical diagnosis of piriformis myositis is difficult, as it can present with hip pain and mimic sacroilitis or sciatica. Physical examination may be difficult as it is a deep infection and palpation may be inconclusive. We describe the case of a 19-year-old man who presented with fever and right hip pain due to piriformis muscle infection by *Brucella melitensis*.

## Case presentation

A 19-year-old Caucasian man presented to our emergency department complaining of right hip pain and fever. Our patient had been in excellent health until pain developed three days before his presentation. A day before admission, his temperature rose to 38.5°C, with rigors and sweating. He had no history of tobacco use, alcohol use, intravenous drug use, or any other factors for HIV infection. Our patient's medical history was unremarkable. A physical examination revealed painful hip movement and a positive straight leg raise sign, without any other significant abnormalities. Further investigation was deemed necessary and our patient was subsequently admitted to the hospital.

Results of a hip X-ray were unremarkable. Laboratory tests revealed a white blood cell count of 14,900 cells/mm^3 ^(normal 4000 to 11,000 cells/mm^3^) with 70% neutrophils (normal 50% to 70%). He also had elevated inflammatory markers with erythrocyte sedimentation rate (ESR) 120 mm/1 hour (normal: 0 to 20 mm/1 hour) and a C-reactive protein (CRP) level of 16.76 mg/dL (normal: <0.8 mg/dL). His creatine phosphokinase (CPK) level was 52 IU/L (normal: <190 IU/L) and lactate dehydrogenase (LDH) level was 366 IU/L (normal 120 to 230 IU/L). Specimens of blood and urine were obtained for culture. The same day, he was submitted to a computed tomography (CT) scan of the abdomen, which did not show any significant abnormality.

A pelvic magnetic resonance imaging (MRI) scan was performed two days later with a 1 Tesla Gyroscan Intera scanner. Imaging included spin-echo (SE) T1-weighted imaging, short tau inversion recovery (STIR), and SE T1-weighted imaging fat suppression with intravenous gadolinium contrast injection (FAT-SAT+GD) images in the axial plane and SE T1-weighted imaging, TSE T2-weighted imaging, STIR, and T1-weighted imaging SE FAT-SAT+GD in the coronal lane.

**Figure 1:**
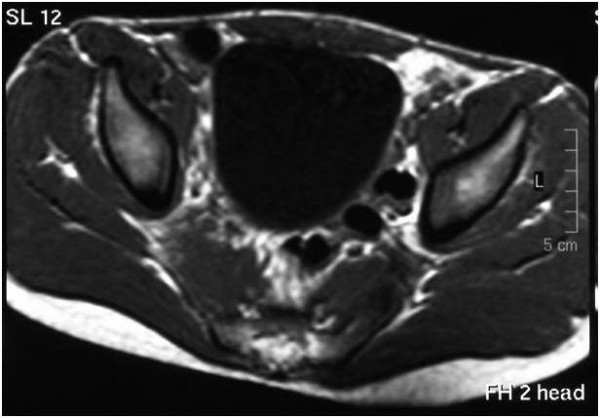
**Axial spin-echo T1-weighted image; MRI scan taken at the time of antibiotic treatment initiation**. The right piriformis muscle is enlarged. The surrounding fat planes are intact.

**Figure 2:**
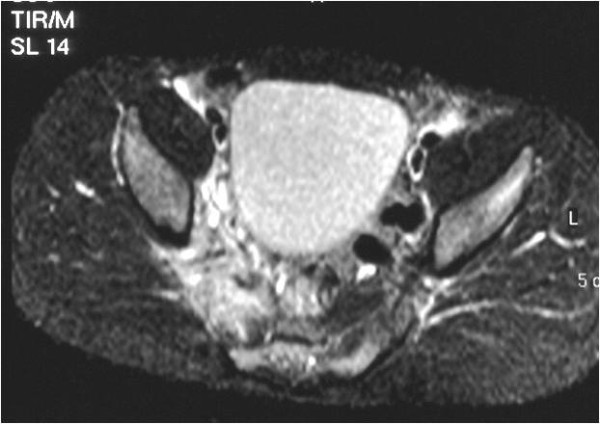
**Axial short tau inversion recovery (STIR) image; MRI scan taken at the time of antibiotic treatment initiation**. The right piriformis muscle is enlarged, bulging anteriorly, with high signal intensity within and loss of definition of its muscle striations, consistent with edema. Compare to the normal piriformis muscle on the left.

The examination revealed a markedly enlarged right piriformis muscle on the T1-weighted image (Figure 1). On the STIR images there was an abnormally high signal intensity in the muscle, suggestive of edema (Figure 2).

**Figure 3:**
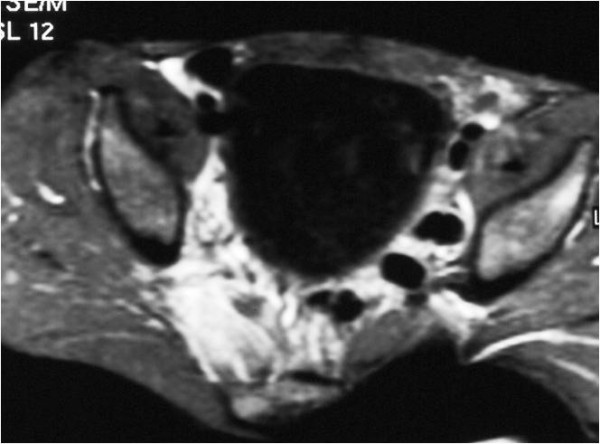
**Axial spin-echo T1-weighted image with fat suppression with intravenous gadolinium contrast injection; MRI scan taken at the time of antibiotic treatment initiation**. The right piriformis muscle enhances avidly after intravenous gadolinium, due to inflammation. Inflammatory changes are also depicted in the adjacent soft tissues of the pelvis.

After gadolinium administration there was widespread pathological enhancement, consistent with the presence of myositis. Inflammatory changes were also depicted in the adjacent soft tissues, spreading along the fascial planes (Figure 3).

Serology with a standard tube agglutination test revealed a titer of >1/1280 for *B melitensis*. Two blood culture tests were also positive for *B melitensis*. Antibiotic treatment for brucellosis was initiated, with doxycycline 100 mg twice daily, rifampin 900 mg daily and ciprofloxacin 500 mg twice daily.

**Figure 4:**
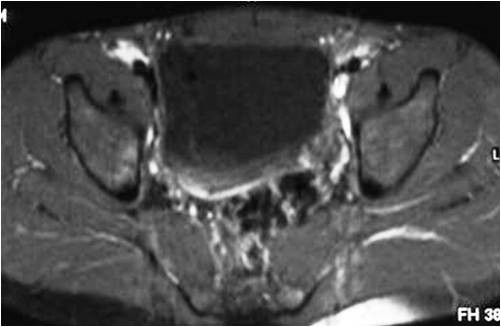
**Follow-up MRI scan (6 months after antibiotic treatment initiation)**. On this axial spin-echo T1-weighted fat suppression with intravenous gadolinium contrast injection, image the right piriformis muscle has normal dimensions and signal intensity compared to the left piriformis muscle. There is no evidence of abnormal contrast medium uptake.

Then, three days after the initial MRI scan our patient was submitted for a bone scintigram, which showed an increased uptake of radiopharmaceutical agent in the right hip, as well as high osteoblastic activity in the right sacroiliac joint. Our patient was dismissed from hospital three weeks later. After six months of antibiotic treatment our patient was asymptomatic. At that time he was submitted to a follow-up MRI, which exhibited complete resolution of the previous findings (Figure 4). Our patient has remained asymptomatic to date.

## Discussion

Myositis is a rare bacterial muscle infection mainly due to *Staphylococcus *and *Streptococcus*. *Brucella *spp. are also a known cause of myositis, along with an extensive list of other pathogens [7]. Infection of the piriformis muscle has been reported six times previously in the literature [1-5], with two of the reported cases sharing a similar obstetric history [3,5]. There have been reports of *Brucella *infection involving various unusual muscle groups [8]. However, involvement of the piriformis muscle in brucellosis, as first presentation, has not been previously reported in the literature.

Piriformis pathology is a known but rare cause of hip pain and sciatica. This is due to its anatomic affinities, with the sciatic nerve closely related to the piriformis muscle, as it exits the pelvis through the greater sciatic notch. In this way a swollen muscle will impinge on the nerve, as it is encased between the piriformis and superior gemellus muscle. It is also important to address the fact that piriformis infections will finally extend to the retrofascial compartment and can become a source of confusion for the clinician, as the problem would seem worse at a more distant site than at the site of origin [3]. This makes the clinical diagnosis of piriformis myositis difficult, as it can present with hip pain and mimic sacroilitis or sciatica. Physical examination may be difficult as this is a deep infection, and palpation alone may be inconclusive.

CT in an emergency situation with intravenous contrast may be helpful in cases of frank pyomyositis, as ring-enhancing lesions will be detected within the muscle. The usefulness of MRI far outweighs CT in cases of uncomplicated myositis, because CT findings will be subtle, inconclusive and may be overlooked. This was true for our case. In cases where CT or MRI are not readily available, ultrasonography may have a limited role in helping reach a diagnosis as it is operator dependent, requiring some degree of expertise. Other possible drawbacks of ultrasonography could be the inability of the patient to cooperate sufficiently due to pain and the inability to determine whether there is concomitant bony involvement [9].

In terms of disease course, myositis is known to have three distinct phases, as described by Chiedozi [10]. During the first phase inflammation is minimal. The muscle becomes hardened, with mild leukocytosis with no evidence of pus. After two to three weeks of the initial symptoms the inflammation increases with evidence of purulence. The third phase is characterized by signs of systemic toxicity. Our patient presented at the beginning of the first phase and use of MRI greatly contributed to the prompt diagnosis. This was important as delayed diagnosis can result in increased morbidity and mortality, which are known to be up to 10% [10]. In cases where there is inflammation only (myositis) and/or minimal abscess collections, antibiotics alone will be the treatment of choice. Serological markers, blood cultures or cultures of the abscess fluid will help establish the diagnosis of the causative organism. In our case both serology and blood culture results were positive. The presence of inflammation alone with no evidence of abscess formation meant that our patient could be treated conservatively with antibiotics. If the diagnosis had been delayed there would have been a need to perform either percutaneous image-guided drainage or surgery [1]. Surgery could involve tenotomy of the piriformis tendon at its tendinous junction, near the greater trochanter, in order to relieve the sciatic nerve, as has been described in the literature [1].

The results of our case point to a rare disease entity, which may mimic other more common diseases, due symptoms around the hip joint and/or sacroiliac joint, which are both piriformis muscles attachments. The irritation of the adjacent sciatic nerve may mimic disk prolapse at the level of the lumbar spine and the combination of fever may drive the clinician to think of spondylodiscitis.

Confirmation of the diagnosis relies upon MRI, which is the most sensitive imaging modality and can depict piriformis muscle involvement in its early stages [3]. Of course imaging alone is not specific for the causative organism. Serology and blood cultures will only identify the causative organism.

## Conclusion

To the best of our knowledge, this is the first case of piriformis myositis due to *Brucella *infection reported in the literature. In suspected cases an MRI scan of the pelvis is of paramount importance in promptly reaching the diagnosis.

## Consent

Written informed consent was obtained from the patient for publication of this case report and any accompanying images. A copy of the written consent is available for review by the Editor-in-Chief of this journal.

## Competing interests

The authors declare that they have no competing interests.

## Authors' contributions

PK contributed to the conception and design of the manuscript, manuscript preparation and review, literature research and reviewed medical imaging. MM contributed to the conception and design of the manuscript, helped draft part of the manuscript and review of the manuscript. AL helped draft part of the manuscript and review of the manuscript. OR helped with the manuscript review and literature research. ES reviewed the medical imaging, supervised and contributed to the manuscript preparation and review. All authors read and approved the final manuscript.
